# Risk assessment of earthquake network public opinion based on global search BP neural network

**DOI:** 10.1371/journal.pone.0212839

**Published:** 2019-03-07

**Authors:** Xing Huang, Huidong Jin, Yu Zhang

**Affiliations:** 1 School of Management, Southwest University of Science and Technology, Mianyang, China; 2 CSIRO Data61, Canberra ACT, Australia; Pablo de Olavide University, SPAIN

## Abstract

**Background:**

The article proposes a network public opinion risk assessment model for earthquake disasters, which can provide an effective support for emergency departments of China.

**Method:**

It uses the accelerated genetic algorithm (AGA) to improve BP neural network. The main contents: This article selects 10 indexes by using the methods of the principal component analysis (PCA) and cumulative contribution (CC) to assess the risk of the earthquake network public opinion. The article designs a BP algorithm to measure the risk degree of the earthquake network public opinion and uses AGA to improve the BP model for parameter optimization.

**Results:**

The experiment results of the improved BP model shows that its global error is 7.12×10, and the error is reduced to 22.35%, which showed the improving BP model has advantages in convergence speed and evaluation accuracy.

**Conclusion:**

The risk assessment method of network public opinion can be used in the practice of earthquake disaster decision.

## Introduction

By June 2018, the number of China’s internet population reached 802 million, and internet popularity is up to 54.3%. The internet popularity provided a convenient for internet user to express their attitudes and views, and their attitudes and views might come into being network public opinion. The opinion spread through network media, more or less, that it can promote or impede the development of the situation, and then the network public opinion of earthquake disaster was a representative of many events. Especially the network public opinion, driven by complex causes, often presents a large number of negative effects, which may cause serious secondary or derivative disasters.

Numerous practices showed: if the risk of network public opinion under the background of earthquake disasters could be scientifically evaluated, it may help both offer a large of information from the disaster area and increase the speed of response. So far, some studies mainly focused on the hot degree of network public opinion, spread rules, risk management and assessment methods. Firstly, on the research of the network public opinion hot degree, researchers mainly focused on the indexes of hot degree of network public opinion. For instance, Zhang Y W (2010) [1]studied the evaluation indexes and weight of network public opinion on unconventional emergencies; Fang Q (2016) [2] took WeChat as the object, and he thought that the hot degree of the WeChat public number is that related to the theme, push time and title character, but it has a weak correlation with the push frequency; Zhao L *et al*(2012)[3]studied the interaction between evolution of emergencies, the spread of public opinion and official media; Cao X Y (2014) [4] studied the correspondence between the hot degree of network public opinion and the risk level of emergency events, and also put forward some strategies for coping with emergencies according to the "consistency" or "inconsistency" of corresponding relations; Wang H J (2012) [5] researched the monitoring problem of the hot degree of network public opinion according to optimization theory; Wei D Z (2017) [6]took the similarity of the content on news webpage and the analysis of page links as the basis for the calculation of the topic hot degree, and put forward a model of hot topic discovery to base on time series; Zhai X F (2015)[7] analyzed the hierarchical rules of transmit micro-blog and defined a new calculation method of hot degree indexes around transmit depth and breadth indexes; Jiao C (2012)[8] put forward the Poisson distribution model of the hot degree distribution for the independent network; Fu X H (2016)[9]simulated on the hot point time of network by clustering algorithm and simulation model. Secondly, on the research of the network public opinion spread rules, Wang Z Y (2017)[10]studied the law of public opinion spreading, management and restrictions through government intervention; Huo L (2011)[11] found an interaction model, which showed the official behavior was how to influence the spread of public opinion spread in emergency events; Qi J Y (2017)[12]studied the interconnection mechanism of network public opinion under the background of emergencies, and thought that the coordination degree of the network public opinion is proportional to the social influence; Kang W (2012) [13] took the “11·16 school bus accident” as an example, and studied the spread influence of network public opinion to the path, speed and scope of information spread. Thirdly, on the research of network public opinion risk management, Zhang Y L (2012)[14] put forward network public opinion risk indexes according to emergency cycle; Lu Z F (2016) [15] took the hot events of public opinion as validation objects, and studied the risk degree of various hot events of public opinion; Lan Y X (2014)[16] built the theoretical framework of public opinion risk, and calculated the risk level basing on the quantitative analysis method. Fourthly, on the research methods of network public opinion risk assessment, Lebensztayn E R (2013)[17] built a Markov model for the spread of mass rumors; Hong L (2011) [18] researched the discovery mode of the hot degree of network public opinion by support vector machine (SVM); Gao H *et al*(2014) [19] analyzed the various influences on risk of network public opinion; Lu Z F *et al* (2016)[20] adopted factor-cluster analysis method to solve the question of network public opinion classification.

In summary, two questions need be solved. One question was the assessment indexes of network public opinion. Current studies mainly preferred external cause of events, for example, the social attributes included the amounts of reload, comments and original essays, and the physical attributes of the event itself included motivation, sensitivity and promotion factors, which often were overlooked. The external factor indexes were mostly gained from the quantitative indexes, while some kinds of qualitative indexes were ignored. For example, the key uncertain indexes of network public opinion under the emergency were ignored. The other aspect was the lack the researches of comprehensive risk evaluation in network public opinion under earthquake disaster, and current studies are mostly around the distribution, linear fitting and prediction of hot spots. A few studies focused monitoring indexes and evaluation approaches, but these monitoring indexes were mostly gotten from the social attributes, and barely involved in the physical attributes indexes. Furthermore, the evaluation method is relatively simple in many articles, and many researchers neglected the nonlinear, high dimensional and non-normal problems of indexes data, which may bring a significant deviation between result and the actual situation. According to the characteristics of network public opinion, the BP method can solve the question of risk assessment. BP neural network is a negative gradient optimization algorithm, which has the advantages of adaptive, self-organizing, fault-tolerant and robustness, and is easy to compile on the computer. It can effectively remove the limitations of multi-index nonlinear, high dimension and non-normal evaluation of the earthquake network public opinion risk, moreover it can also ensure the reliability of the assessment results by approximating any square-integral nonlinear continuous function with the accuracy of arbitrary mean squared deviation. The article will propose a set of risk assessment indexes, which can cover the whole public opinion assessment cycle from both aspects of physical properties and social attributes. It will adopt the method of BP neural network to evaluate the risk of network public opinion under background of earthquake disaster, and use AGA model to improve BP neural network for increasing the reliability of the assessment results.

## The risk indexes of earthquake network public opinion

### Determination of index

The spread of network public opinion requires unusually a certain process, and the occurrence and expansion of risk are mainly driven by public sentiment carrier, subject and itself. Network media is the carrier of earthquake public opinion, and it belongs to the condition factor. Network users are the main part of the earthquake public opinion, which is the promoting factor. Earthquake disaster belongs to the induction factor. The condition factor and the promoting factors belong to the social attribute of the diffusion and propagation) of the earthquake network public opinion, and the induction factors are the physical, and then the sensitivity factor can lead to a driving effect to the network public opinion. On the design of risk indexes, this study focused on both the physical and social attributes of the earthquake network public opinion.

**(1)The physical attributes of the earthquake network public opinion.** It’s determined by the damage degree of the earthquake. Generally speaking, the damage degree is the heavier, and the social influence also is the higher, and then the stronger the ability of the diffusion of the earthquake network public opinion. There are two types of indexes for assessment the physical attributes of the earthquake network public opinion, including damage level and disaster degree. The damage level can be measured by the indexes of earthquake magnitude and central intensity, and the indexes of disaster degree can be measured by the fatalities, affected area and property loss, etc.

**(2) The social attributes of earthquake network public opinion.** It can be measured by indexes of emergency response ability, network media force and diffusivity ability. The emergency response ability has a direct impact to relieve disaster losses and negative emotions of network users. Emergency response ability mainly includes the emergency resources, reaction ability of government, ability of monitoring and early warning, etc. The network media force mainly consists of the number of original topics, comments and forwarding, etc. The diffusivity ability consists of the ratio of the clicks amount to responses and the change rate of the original topics.

This article put forward firstly 13 indexes from physical properties and social attributes. To verify all indexes of independence, correlation and representative, this article adopted the method of expert scoring to gain the initial data and used Cronbach Alpha to measure the reliability, and used Kaiser-Meyer-Olkin (KMO) and Bartlett sphere tested the availability of the questionnaire. The result showed Cronbach`s Alpha = 0.871, so the questionnaire, be proved, is reliable; where, the KMO = 0.743, and the Bartlett spherical = 0.000, which indicated that the questionnaire was quite available. The result showed P < 0.001 in the Bartlett sphere test, and it suggested that there were some repeated interpretation indexes in 13 indexes, so the article deleted the index of “the affected scope”, its relevancy > 0.6. To test the correlation of indexes, and the article deleted these indexes, which correlation <0.651, such as central intensity of earthquake. Secondly, a principal component analytical method was used to find the explanation ability of all indexes. Now there were two results be needed, including the contribution rate and the cumulative contribution rate of each component. The criterion of judgment was: looking on the cumulative contribution rate of 85%+ as the final evaluation indexes (as shown in Table 1).

**Table 1:** Network public opinion risk indexes.

Fist-level indexes	Second-level indexes	Third-level indexes	Data
**Physical attributes**	Damage level	Earthquake magnitude	According to the official data
		Incidence of secondary disaster	According to historical data to estimate the risk of the secondary disaster, the occurrence probability of M4+ aftershocks: main shock magnitudes is M6, incidence of secondary disaster is 30%; Main Shock is M7, incidence of secondary disaster is60%, main shock magnitudes is above M8,incidence of secondary disaster is 90%.
	Disaster degree	Casualties	According to the official data
		Property loss	According to the official data
**Social attributes**	Emergency response ability	Emergency resources	Valuation: Stronger(5),Strong(4),General(3),Weak(2),Weaker(1)
		Monitoring and early warning	Valuation:Stronger(5),Strong(4),General(3),Weak(2),Weaker(1)
		Comprehensive satisfactory degree of the victims	Valuation:Stronger(5),Strong(4),General(3),Weak(2),Weaker(1)
	Network media force	Posts	The posts of mainstream media during limited time
		Comments	The comments of mainstream media during limited time
		forwarding	The forwarding of mainstream media during limited time

In Table 1, the data of earthquake emergency response ability was determined by the experts, who adopted the Delphi method. The data of the network force came from the Beijing Qingbo big data technology co. Ltd through gaining the data of posts, comments and forwarding around 6 network medias, such as Wechat, MicroBlog, web pages, Newsarticles, the client and the BBS[21]. The data came from the qualitative score of experts, public opinion data system and the official website of china. The data, including the amount of original topic, forwarding amount and commended amount, was counted within 30 days after the earthquake. In addition, the raw data for this manuscript comes from the research team's questionnaires, interviews and measurements.

### The risk level

Risk level standard of the earthquake network public opinion consisted of two parts: setting the risk level and confirming the interval value of each index. This article referred to international conventions and china studies, then divided the risk level of earthquake network public opinion standards into 4 levels: v = {Ⅳ,Ⅲ, Ⅱ, Ⅰ} = {higher risk, high risk, general risk, low risk}; Valuation: “higher risk” for 4, “high risk” for 3, “general risk” for 2 and “low risk” for 1. For confirming the interval value of each index, the article choices “The Chinese public events database” made by Shanghai Jiaotong University public opinion research laboratory as the date source[22]. The study selects 150 cases, which occurs after 21 century and the seismic grade lies in 3~9. The qualitative indicators assigned according to Table 1 and got data through the questionnaires, and the quantitative indexes assigned according to the objective data in the specific period, among them, the earthquake magnitude, casualties and property loss risk standard according to the “act of China earthquake emergency preparedness”. The risk level of rest indexes is determined on the basis of the research result of Jiang J C (1996)[23]. In addition, the data of earthquake network public opinion indexes are usually uncertainty, and the risk level standard of each index is difficult to be determined, so it is more reasonable to use fuzzy interval numbers to express the risk level of each index. For that reason, the four points risk partition method is used to determine the risk level standard of earthquake network public opinion, the specific approach: firstly, arranging the value of *z(i)* by ascending order, look on *z(i)*_*max*_ as the uppermost value, and *z(i)*_*min*_ is regards as the lowest limit value. Median *z(i)*_*med*_ is regards as the general risk value. Secondly, finding out the median z^*^(i)_*med*_ from *z(i)*_*min*_ and *z(i)*_*med*_, and the median z**(i)_*med*_ from *z(i)*_*med*_ and *z(i)*_*max*_, as shown in Table 2.

**Table 2:** Earthquake network public opinion risk rating standard.

Risk level	Ⅰ	Ⅱ	Ⅲ	Ⅳ
**Interval value**	(-*∞*, z^*^(i)_*med*_)	[z^*^(i)_*med*_, *z(i)*_*med*_]	[*z(i)*_*med*_, z^**^(i)_*med*_]	[z^**^(i)_*med*_, +*∞*]

Furthermore, according to the Table 2, we calculated the standard interval value of each indicator of in different risk level, as shown in Table 3.

**Table 3:** Earthquake network public opinion risk rating standard.

Index	Low risk = 1	General risk = 2	High risk = 3	Higher risk = 4
	Ⅰ	Ⅱ	Ⅲ	Ⅳ
**earthquake magnitude**	[1,3]	[3,5]	[5,7]	[7, +*∞*]
**incidence of secondary disaster**	[0,0.2]	[0.2,0.45]	[0.45,0.65]	[0.65,1]
**fatalities**	[0,10]	[10,50]	[50,300]	[300, +*∞*]
**property loss ($100 million)**	[0, 0.01 *GDP]	[0.01 *GDP, 0.06*GDP]	[0.06*GDP, 0.1*GDP]	[0.1*GDP, +*∞*]
**emergency resources**	[4,5]	[3,4]	[2,3]	[1,2]
**monitoring and early warning**	[4,5]	[3,4]	[2,3]	[1,2]
**comprehensive satisfactory degree of the victims**	[4,5]	[3,4]	[2,3]	[1,2]
**original topic**	(-*∞*,40000)	[40000,100000]	[100000,500000]	[500000, +*∞*]
**Number of Comment**	(-*∞*,240000)	[240000,600000]	[600000,3000000]	[3000000, +*∞*]
**forwarding amount**	(-*∞*,120000)	[120000,300000]	[300000,1500000]	[1500000, +*∞*]

## The AGA-BP assessment model

The slow learning speed of BP neural network and the existence of local minimum problems will affect the network's predication ability [24–26]. To solve these problems, the AGA was used to improve the conventional BP neural network, because the AGA can optimize the network parameters of BP. After that the optimized parameters will be treated as the initial value of the BP algorithm. It can effectively enhance BP neural network's extrapolation ability, as well as preventing the network from entering partial circulation.

### Steps to build the AGA-BP model

**Fig 1:**
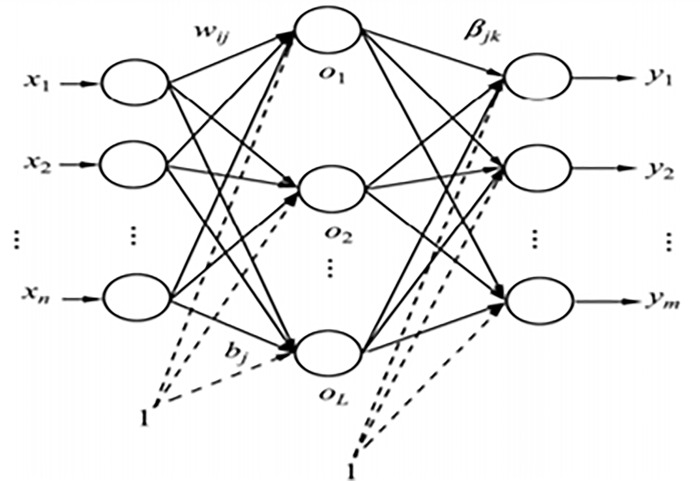
BP neural network model.

In this article, the BP neural network is consisted of three layers (Liu C X *et al*, 2017)[27]: the first layer is the data input layer, the second layer is the hidden layer, and the third layer is the data output layer. Each layer is connected by some nodes, which are usually the place where data is input and output (As shown Fig 1).

Here is no strict restriction on the number of the output layer nodes *n* and that for the hidden layer node *m*, as the existing three-layer BP neural network can approximate to the mapping between input layer and output layer by arbitrary precision. According to experience, the number of nodes of the hidden layer *m* is relatively small; if the *m* relatively bigger, the generalization capability and training speed of BP network would be affected. Generally speaking, the range of value *m* is generally controlled in [*n*, *2n+1*] and as small as possible under the conditions of permissible precision. In this study, three layers of BP neural networks were selected, and the number of nodes at each level was *n*: *n*: *1*, in which *n =* 3. The symbols of the model are defined, as follows:

*h*: the input layer neuron.
*i*: the hidden neuron.
*j*: the output neuron.
*θ*_*i*_: the threshold of the hidden neuron.
*θ*_*j*_: the threshold of the output neuron.
*w*_*hi*_:the connection weights between the Input layer neuron and the hidden neuron
*w*_*ij*_:the connection weights between the hidden neuron and the output neuron
*x*: input point
*y*: output point
**Step 1:** Acquiring random sample points of indexes. In the BP neural network training, at first, it is necessary to generate without dimensionless of random sample data of the risk grade of network public opinion under earthquake disaster. After training the optimal parameters through the random sample data, then the BP model is tested with the actual sample data again. In the acquisition of random sample data, this study referred to the research results of Wang S (2006), and used the uniform random number generated the sample value of indexes in the change rang of indexes, which are expressed by x*(*k*,*j*). While the standard value of risk level of network public opinion under earthquake disaster is expressed by *y*(*k*) = *i*. In order to fully reflect the information of the boundary value of each index, the boundary value of every index is used only one. The arithmetic mean of two risk grade values related to the boundary value is taken from the risk rank value, so that the sample series, which is expressed by {x*(*k*,*j*),*y*(*k*)},*k* = 1~*nk*,*j* = 1~*nj*, in which the mean of *nk* is the number of samples, of the risk assessment standard of the earthquake disaster network public opinion can be obtained. Further, the study need eliminate the dimension of indicators to make the evaluation model universality. The dimensionless of the indexes is as follows,

$$x\left(k,j\right)=x^{*}(k,j)/x_{\max}(j)(k=1∼nk,j=1∼nj)$$ (1)

$$a\left(i,j\right)=a^{*}(i,j)/x_{\max}(j)(i=1∼ni,j=1∼nj)$$ (2)

$$b\left(i,j\right)=b^{*}(i,j)/x_{\max}(j)(i=1∼ni,j=1∼nj)$$ (3)

**Step 2:** Initialization of BP model. The samples values of the input and output of the earthquake network public opinion for machine learning are set as {*x*_*hk*_,*d*_*k*_|*h* = 1,2,…,*n*;*k* = 1,2,…,*N*}. What’s more the connection weight and the threshold value between the nodes are given on the (-1, 1) interval.
**Step 3:** For *k = 1*. Inputs and outputs of each layer {*x*_*hk*_,*d*_*k*_} are provided to the network, where (*h = 1*,*2*,*…*,*n; k = 1*,*2*,*…*,*N*)
**Step 4**: Calculate the input *x*_*i*_ and output *y*_*i*_ of each nodes of the hidden layer. Output the input of layer node *x*_*j*_ and the output *y*_*i*_,

$$x_{i}=\sum_{h=1}^{n}w_{hi}x_{hk}+\theta_{i},y_{i}=1/(1+e^{-x_{i}}),(i=1,2,\ldots ,n)$$ (4)

$$x_{j}=\sum_{i=1}^{n}w_{it}y_{i}+\theta_{j},y_{j}=1/(1+e^{-x_{t}})$$ (5)

**Step 5:** Calculate the output layer node receive the change rate of the single sample error *E*_*k*_ = 0.5(*y*_*j*_−*d*_*k*_)^2^ with the change of the total input and the hidden layer nodes receives the change rate of single sample error *E*_*k*_ with the change of total input,

$$\frac{\partial E_{k}}{\partial x_{j}}=y_{i}(1-y_{i})(y_{i}-d_{k})$$ (6)

$$\frac{\partial E_{k}}{\partial x_{i}}=y_{i}(1-y_{i})(\frac{\partial E_{k}}{\partial x_{j}}w_{ij}),(i=1,2,\ldots ,n)$$ (7)

**Step 6:** amended the weight and threshold of each connection,

$$w_{ij}^{m+1}=w_{ij}^{m}-\eta y_{i}\frac{\partial E_{k}}{\partial x_{j}}+a(w_{ij}^{m}-w_{ij}^{m-1}),\theta_{j}^{m+1}=\theta_{j}^{m}-\eta \frac{\partial E_{k}}{\partial x_{j}}+a(\theta_{j}^{m}-\theta_{j}^{m-1})$$ (8)

$$w_{hi}^{m+1}=w_{hi}^{m}-\eta x_{hk}\frac{\partial E_{k}}{\partial x_{i}}+a(w_{hi}^{m}-w_{hi}^{m-1}),\theta_{i}^{m+1}=\theta_{i}^{m}-\eta \frac{\partial E_{k}}{\partial x_{i}}+a(\theta_{i}^{m}-\theta_{i}^{m-1})$$ (9)

Where, *m*: number of correction. *η*: learning rates and *η*∈(0,1). *α*: Momentum factor and *α*∈(0,1).

**Step 7:** For *k = k+1*, go to step 3 till *N* sample points are trained, after that go to step 9.
**Step 8:** Go to step 2, a new round of learning is performed until the network global error function $E=\sum_{k=1}^{N}E_{k}=\sum_{k=1}^{N}{\left(y_{j}-d_{k}\right)}^{2}/2$ is less than a pre-set value or the number of studies is greater than the pre-set value. It need to determine the optimal value of *θ*_*i*_、 *θ*_*j*_、 *w*_*hi*_ and *w*_*ij*_ of the BP network to minimize the global error function of Eq (7), and at the same time, to stabilize the weight and threshold of network connection at all levels. What’s worse the slow learning speed of BP neural network and the existence of local minimum problems will affect the network's extrapolation ability to a great extent. This study uses AGA to optimize BP neural network parameters, which are looked as the initial value of BP neural network to avoid the shortage of BP neural network. The optimization steps are as follows:

1. **Constructing the change interval of BP neural network parameters.** Set *c*_*j*_ is the value of any parameter of the network when the training network of BP algorithm shows the convergence speed at a slow pace. Change interval of *c*_*j*_ is [*a*_*i*_,*b*_*j*_], in which *a*_*j*_ = *c*_*j*_−*d*|*c*_*j*_|,*b*_*j*_ = *c*_*j*_+*d*|*c*_*j*_|, *d* is a positive constant.
2. **Coding the BP neural network parameter.** *e* refers to the length of the code, which divide the interval [*a*_*j*_,*b*_*j*_] into 2^*e*^−1 subintervals, therefore, the entire network parameter variation space is discredited into grid points (2^*e*^)^*p*^. Among them: *p* = 2*n*^2^+*n*+1. Each grid point is seen as an individual, which corresponds to a possible value state of *p* parameters, which is represented by the *e* bit binary number of *p*. Therefore, the network parameters, grid points, individuals and binary numbers of *p* correspond to each other.
3. **The random generation of the initial parent group, and the evaluation of the individual fitness of the parent.** *n* points are randomly selected from the above (2^*e*^)^*p*^ grid points as the initial parent group. The network of global error function value *E*_*i*_ is got with the *i-*th individual getting into Eq (7). The smaller the *E*_*i*_ is, the stronger the individual ability.
4. **The Selection, hybridization of the parent.** The parent is ranked according to the optimal function value, and the first few individuals in the first order are called excellent individuals. Construct the inverse function *p*_*i*_ against *E*_*i*_, for *p*_*i*_>0, *p*_1_+*p*_2_+⋯+*p*_*n*_ = 1, totally *i* were selected with *p*_*i*_ probability. Thus, two groups, which contain *n* individuals, were selected, and two pairs of individuals were randomly paired into *n* pairs, and then the binary array of parents was exchanged for any value of the binary array, and the two groups of offspring individuals were obtained.
5. **The variation of offspring individuals.** A group of offspring individual is randomly selected from the hybrids of the parent. The arbitrary two values of their binary array are flipped by the mutation rate, which means the original value will be changed from 0 to 1, vice versa.
6. **The iteration.** The offspring individual of *n* from step6 is seen as the new parent. The algorithm is transferred to the parent individual fitness evaluation step, and entered the next generation evolution process.
7. **Cyclical acceleration.** Using the variation range of the excellent individual parameters are produced by the first and second evolutionary iterations that is regarded as the new initial range of parameters, then the algorithm enters the network parameter coding step, the process always is repeated following as step 2. Like this again and again, the parameter change interval of the excellent individual will gradually shrink, and the optimal distance will be closer and closer until the given acceleration times are reached.

**Step 9:** Set the untested evaluative value of each single evaluation model in *K* period as input sample. That is, inputting the network that is been completed. The output value of network, by the inverse treatment of normalization, just is regards as the combination evaluation value *F*_*k*_.

### Control parameter setting

In the AGA-BP model algorithm, the control parameters are set as follows:

The learning rate of BP neural network: *η* = 0.1.
The momentum coefficient of BP neural network: *a* = 0.1.
The coding length of AGA: *e* = 10.
The rate of AGA variation: *p*_*m*_ = 1.0.
The number of individual parent: *q* = 300.
The number of excellent individual: *s* = 10.

## The application of example

### Selection of sample data

The samples of this study came from “*earthquake cases in China*”, in which 6 earthquakes cases be selected (as shown in Table 4). Earthquake magnitude, casualties and property loss came from the official website of China. The validation has two stages: firstly, the study uses the random data to train AGA-BP model to obtain the optimal parameters, and compared the AGA-BP with the BP neural network to observe their training precision. At the second stage, 6 samples data is loaded into the AGA-BP model to evaluate their risk degree.

**Table 4:** Data of 6 earthquakes occurred in the country.

Serial number	Evaluation indexes									
	Earthquake magnitude	Incidence of secondary disaster	Fatalities	Property loss	Emergency resources	Monitoring and early warning	Comprehensive satisfactory degree of the victims	Original topic	Comments	Forwarding
1	5.7	0.3	416	20	3	3	2	5200	110970	35098
2	6.4	0.25	31	18.98	3	3	2	12390	136708	72390
3	8.0	0.85	443870	8451.4	4	3	3	160876	3245626	978690
4	7.1	0.8	9727	8000	4	4	4	224560	4321567	1608900
5	7.0	0.8	11579	422.6	4	3	3	189678	5789430	997860
6	6.5	0.55	3760	4.6	2	3	2	6900	21000	35067

### Parameters training and assessment of AGA-BP model

#### The training of random sample date in AGA-BP Model

According to earthquake network public opinion risk standard in Table 3 and step 1, and then the risk standard of the 1~31 groups sample data were randomly generated, as shown in Table 5.

**Table 5:** Comparison earthquake net-mediated public sentiment risk assessment random date with AGA-BP results.

Serial number	Evaluation indexes										Risk level	
	Earthquake magnitude	Incidence of secondary disaster	Fatalities	Property loss	Emergency resources	Monitoring and early warning	Comprehensive satisfactory degree of the victims	Original topic	Comments	Forwarding	Standard value	AGA-BPResults
1	3.0	0.12	0	0.12	5	4	5	2354	4106	3298	1	1.002
2	3.0	0.16	1	0.31	5	4	5	6549	120001	2109	1	1.011
3	3.0	0.13	3	0.22	5	4	5	9102	16090	4190	1	1.001
4	3.0	0.17	0	0.15	5	4	4	8100	3768	1789	1	1.004
5	4.0	0.28	13	0.61	3	3	3	40100	250019	130290	2	1.977
6	4.0	0.34	11	0.88	3	3	3	51089	310988	160901	2	2.006
7	4.5	0.41	13	1.28	3	3	3	71000	248907	125890	2	2.010
· · ·	· · ·	· · ·	· · ·	· · ·	· · ·	· · ·	· · ·	· · ·	· · ·	· · ·	· · ·	· · ·
27	6.8	0.55	67	16.69	2	2	2	118903	600897	439809	3	3.031
28	6.0	0.47	52	13.37	2	2	2	158902	310980	389070	3	2.891
29	7.1	0.81	7289	1345.67	1	1	1	789087	5189089	1790800	4	3.899
30	8.0	0.97	32980	7616.24	1	1	1	1378190	3190807	2189070	4	4.008
31	7.7	0.88	23109	3178.99	1	1	1	467190	6678902	2897602	4	4.010

**Fig 2:**
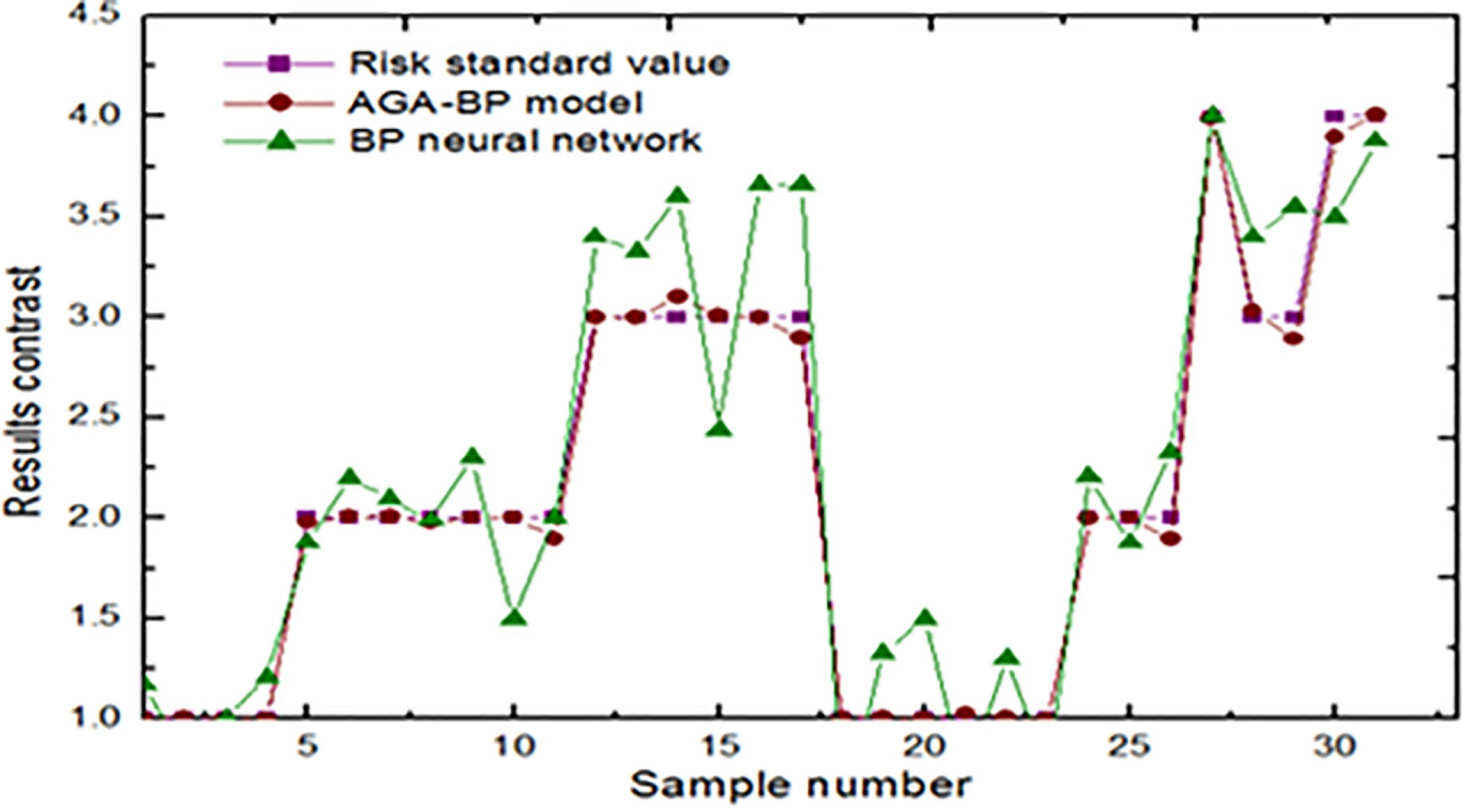
Training accuracy comparison.

Using the formula (1) ~ (3) cope with the sample data to eliminate the difference measure unit of all indexes and then input the normalized sample data into the AGA-BP model to learn and train. The train number is 1,000 times. The optimization acceleration number of the AGA-BP is 4. At last, the calculation results were as Table 5. Further, input the random sample data into BP neural network, and their training accuracy as shown in Fig 2.

The Fig 2 shows: the global error of the AGA-BP evaluation model after 1,000 times training is 0.000712, which meets the convergence requirement. The global error of BP neural network is 0.000917. Compared with the actual risk level, the accuracy of AGA-BP model is significantly higher than that of BP neural network. At this point, the stable weight values and threshold of the AGA-BP model are shown in Table 6.

**Table 6:** The stable weight values and threshold of the AGA-BP model after training1000 times.

Parameter	Hidden Neurons)*i*		
	1	2	3
*w*_1*i*_	-0.3120	-2.1342	-1.6750
*w*_2*i*_	-3.4561	5.1309	1.8674
*w*_2*i*_	-1.3758	2.9879	1.2324
*θ*_*i*_	0.8609	1.3561	-0.2044
*w*_*i*1_	-2.5176		
*w*_*i*2_	3.3256		
*w*_*i*3_	-2.1843		
*θ*_*j*_	-12.3127		

#### Case assessment

**Fig 3:**
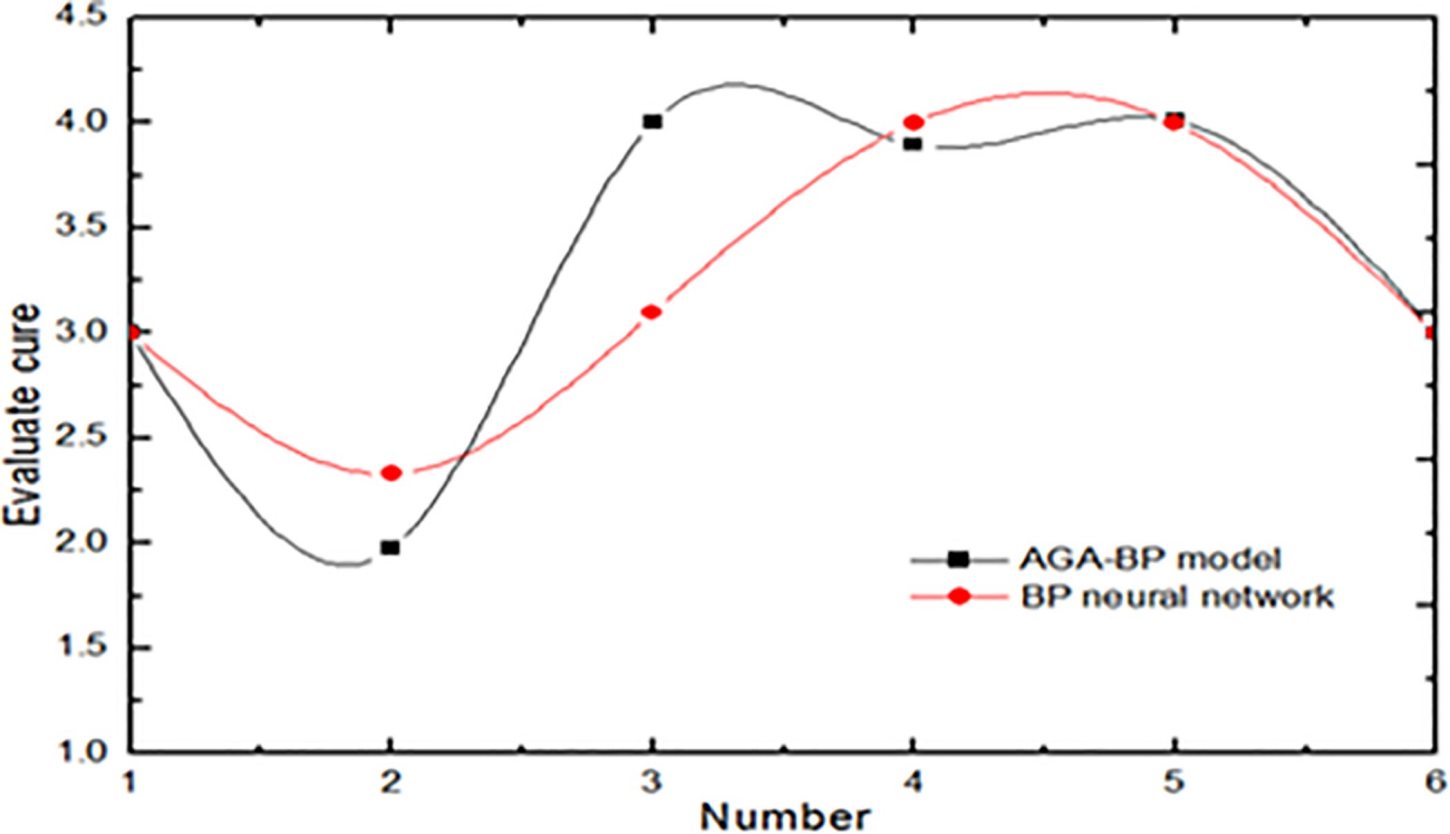
Evaluation curve of AGA-BP and BP.

After 1,000 times training, the threshold and weights values of each layer of AGA-BP model trend a stability, and the accuracy satisfied nearly the requirements, so it shows that the AGA-BP model can evaluate the risk of the earthquake network public opinion. Then the article will evaluate the 4 cases in Table 6 by AGA—BP model, BP neural network and logistic curve to show the advantage of AGA-BP model. Some parameters are set as follows: the train times are 15,000 to the AGA-BP model and BP neural network, the optimal acceleration is 4 times, and the other parameters were constant. The logistic curve still adopts the fitting method for risk assessment. The results are shown in Table 7 and Fig 3.

**Table 7:** Evaluation results of three methods.

	Jiujiang,Jiangxi	Ninger,Yunnan	Wenchuan,Sichuan	Yushu,Qinghai	Ya 'an,Sichuan	Ludian,Yunnan
**AGA-BP model**	3.0002	1.9770	4.0012	3.8964	4.0110	3.0000
**BP neural network**	3.0000	2.3310	3.0978	4.0004	3.9971	2.9963

### Result analysis

The results of the AGA-BP model on the risk assessment show that:

1. Earthquake magnitude, fatalities, property loss, posts, comments and forwarding are the key indicators that affect the risk level of earthquake network public opinion. The training results of the random data shows: there are 4 indexes, including the emergency resources, the capability of monitoring, early warning and comprehensive satisfactory degree of the victims, which have a low influence to the risk level of earthquake network public opinion.
2. The evaluation results of the AGA-BP model are consistent with the actual results and it’s more feasible than BP neural networks. In Table 7, the BP neural network evaluates the Wenchuan earthquake as high risk, obviously, it’s impractical. Actually, though the network public opinion of the Wenchuan earthquake spread rapidly, those numerous negative public opinions were prevented effectively by the government and experts in time.

## Conclusions

The risk evaluation system of earthquake network public opinion is established that is an essential task for improving the efficiency and capacity of emergency response. This study leads to the following conclusions,

**(1) This article put forward the risk monitoring indexes for earthquake network public opinion.** According to appear feature and propagation law of the earthquake network public opinion, the primary risk monitoring indexes were set around the physical and social property. After all indexes were tested around the reliability and validity, the primary indexes were filtered through the principal component analysis (PCA) and rate of cumulative contribution, at last, the article put forward 10 risk evaluation indexes for earthquake network public. The result of verification shows that the 10 indexes can effectively evaluate the risk of earthquake network public opinion.

**(2) The AGA-BP model is superior to BP neural network.** The result of verification shows the convergence speed, parameter optimization and preventing premature convergence of AGA-BP model are all superior to the BP model. So the AGA-BP model can be used in the practice of earthquake network public opinion risk management.

**(3) The accuracy of AGA-BP model is higher than the BP neural network.** Training samples and example verification show that BP neural network can easily get into local optimal, and the accuracy of BP model is lower than the AGA-BP model. In addition, the training time of BP model is also longer than the AGA-BP model.

In the future, authors will research on network public opinion risk prediction is carried out.
